# Tetra­kis(μ-2-phenyl­acetato-κ^2^ *O*:*O*′)bis­{[4-(di­methyl­amino)­pyridine-κ*N* ^1^]cobalt(II)}

**DOI:** 10.1107/S1600536813023258

**Published:** 2013-08-23

**Authors:** Meriem Benslimane, Yasmine Kheira Redjel, Georges Dénès, Hocine Merazig

**Affiliations:** a Unité de Recherche de Chimie de l’Environnement et Moléculaire Structurale, Faculté des Sciences Exactes, Département de Chimie, Université de Constantine 1, 25000 Constantine, Algeria; b Department of Chemistry and Biochemistry, Concordia University, Montréal, Canada

## Abstract

The title compound, [Co_2_(C_8_H_7_O_2_)_4_(C_7_H_10_N_2_)_2_], crystallizes as a centrosymmetric dimer containing two Co^II^ atoms bridged by four bidentate phenyl­acetate ligands in *syn–syn* bridging modes. Each Co^II^ atom is five-coordinated by four O atoms from four different carboxyl­ate ligands and the ring N atom of a 4-(di­methyl­amino)­pyridine unit, generating a distorted square-pyramidal geometry in which the four O atoms form the basal plane and the N atom occupies the axial position. In the crystal, C—H⋯O inter­actions link the dinuclear complex mol­ecules into a three-dimensional network.

## Related literature

For properties of the 4-(di­methyl­amino)­pyridine ligand as a homogeneous catalyst, see: Satgé *et al.* (2004 ▶). For transition metal complexes of 4-(di­methyl­amino)­pyridine which exhibit luminescence properties, see: Araki *et al.* (2005 ▶). For biological and magnetic properties of carb­oxy­lic acid complexes of cobalt(II), see: Cotton *et al.* (1999 ▶). For related centrosymmetric dinuclear cobalt(II) complexes bridged by carboxyl­ates, see: Cui *et al.* (1999 ▶); Catterick & Thornton (1977 ▶).
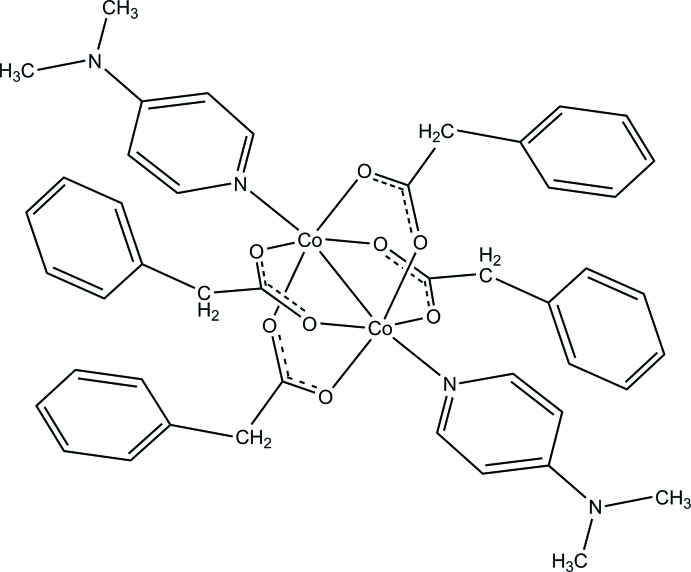


## Experimental

### 

#### Crystal data

[Co_2_(C_8_H_7_O_2_)_4_(C_7_H_10_N_2_)_2_]
*M* *_r_* = 902.74
Triclinic, 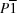

*a* = 8.107 (5) Å
*b* = 11.043 (5) Å
*c* = 12.573 (5) Å
α = 99.766 (5)°
β = 101.878 (5)°
γ = 105.335 (5)°
*V* = 1031.9 (9) Å^3^
*Z* = 1
Mo *K*α radiation
μ = 0.86 mm^−1^
*T* = 150 K
0.15 × 0.10 × 0.01 mm

#### Data collection

Bruker APEXII CCD diffractometer
Absorption correction: multi-scan (*SADABS*; Bruker, 2012 ▶) *T*_min_ = 0.902, *T*_max_ = 0.991
9238 measured reflections
3645 independent reflections
3352 reflections with *I* > 2σ(*I*))
*R*_int_ = 0.019

#### Refinement

*R*[*F* ^2^ > 2σ(*F* ^2^)] = 0.025
*wR*(*F* ^2^) = 0.060
*S* = 1.05
3645 reflections
271 parameters
H-atom parameters constrained
Δρ_max_ = 0.25 e Å^−3^
Δρ_min_ = −0.24 e Å^−3^

### 

Data collection: *APEX2* (Bruker, 2012 ▶); cell refinement: *SAINT* (Bruker, 2012 ▶); data reduction: *SAINT*; program(s) used to solve structure: *SIR92* (Altomare *et al.*, 1993 ▶); program(s) used to refine structure: *SHELXL97* (Sheldrick, 2008 ▶); molecular graphics: *ORTEPIII* (Burnett & Johnson, 1996 ▶) and *ORTEP-3 for Windows* (Farrugia, 2012 ▶); software used to prepare material for publication: *SHELXL97*.

## Supplementary Material

Crystal structure: contains datablock(s) global, I. DOI: 10.1107/S1600536813023258/lr2113sup1.cif

Structure factors: contains datablock(s) I. DOI: 10.1107/S1600536813023258/lr2113Isup2.hkl

Additional supplementary materials:  crystallographic information; 3D view; checkCIF report

## Figures and Tables

**Table 1:** Hydrogen-bond geometry (Å, °)

*D*—H⋯*A*	*D*—H	H⋯*A*	*D*⋯*A*	*D*—H⋯*A*
C6—H6*A*⋯O2^i^	0.96	2.53	3.337 (3)	142
C23—H23⋯O3^ii^	0.93	2.58	3.469 (3)	159

Symmetry codes: (i) 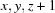; (ii) 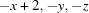.

**Fig. 1.**
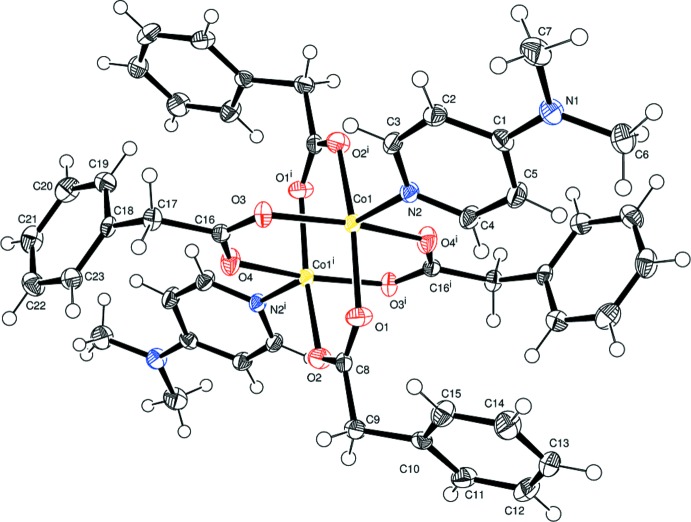
The molecular structure of the title compound. Displacement ellipsoids are drawn at the 50% probability level and H atoms are shown as small spheres of arbitrary radii. Symmetry code, (i):1 - x, -y, -z.

**Fig. 2.**
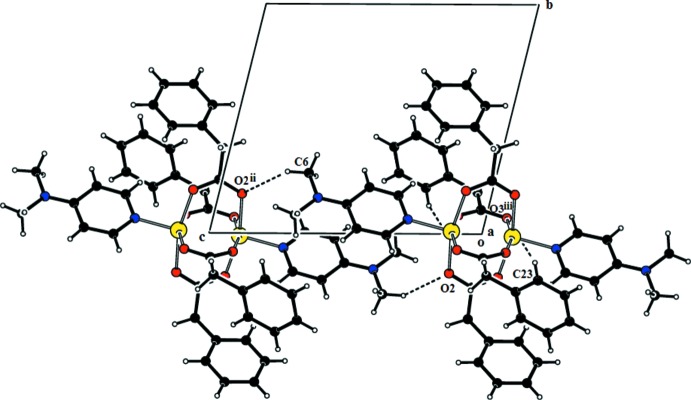
The crystal packing of the title compound, viewed along the c axis. Hydrogen bonds are shown as dashed lines. Symmetry code, (ii): x, y, 1 + z.

**Fig. 3.**
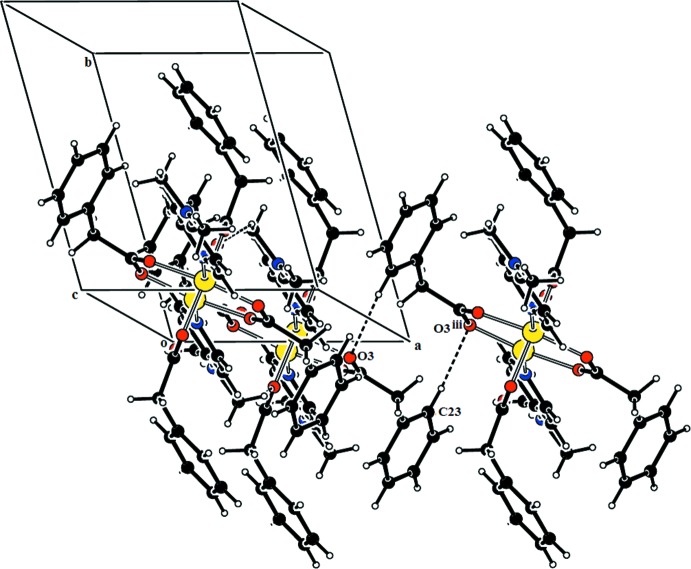
The crystal packing of the title compound, viewed along the a axis. Hydrogen bonds are shown as dashed lines.Symmetry code, (iii):2 - x, -y, -z.

## supplementary crystallographic information

### 1. Comment

The N-heteroaromatic ligand 4-(dimethylamino)pyridine (DMAP) finds use as a homogeneous catalyst in cellulose acylation in the synthesis of biodegradable plastics (Satgé *et al.*, 2004). DMAP is also known to form transition metal complexes which exhibit luminescence properties (Araki *et al.*, 2005). Our interest in cobalt(II) carboxylates with DMAP evolves from their catalytic activity. Moreover, carboxylic acid complexes of cobalt(II) have properties of special interest in the fields of biology and magnetism (Cotton *et al.*, 1999). The coordination chemistry of centrosymmetric dinuclear Co^2+^ complexes bridged by carboxylates has been investigated (Cui *et al.*, 1999, Catterick *et al.*, 1977). In order to explore further the coordination behaviour of the Co^2+^ ion, the title complex, incorporating phenylacetate and DMAP as co-ligand has been prepared and its crystal structure is reported here.

The title molecule is a centrosymmetric dimer with four bidentate phenylacetate groups as bridging ligands between two Co^II^ centres, to each of which a DMAP group is also coordinated, as shown in Fig. 1. The coordination geometry about each Co atom is distorted square pyramidal, with four O atoms from four different carboxylate ligands forming the basal plane and a pyridine N atom occupying the axial position, where the most distorted angle is 101.39 (5)° for O2^i^—Co—N2 [symmetry code: (i) -*x* + 1,-*y*,-*z*]. The interatomic distances of Co—O [2.0224 (12)–2.0628 (16) Å], Co—N2 [2.0460 (16) Å] and Co···Co [2.8019 (12) Å] agree well with the related values recorded for the structures of the analogous pivalate (Cui *et al.*, 1999) and benzoate (Catterick *et al.*, 1977). The Co(II) atom is 0.2286 (2) Å from the mean plane formed by the four equatorial O atoms. On the other hand, the coordinated N2 atom also lies in the same direction, at a distance of 2.2643 (13) Å from the plane. The dihedral angles between the mean planes through the C10—C15 and C18—C23 benzene rings and the DMPA plane are 7.46 (9)° and 72.08 (9)°, respectively. The dihedral angle between the planes trough Co1/O3/O4/C16/Co1^i^/O3^i^/O4^i^/C16^i^ and Co1/O1/O2/C8/Co1^i^/O1^i^/O2^i^/C8^i^ [symmetry code:(i) 1 - *x*, -*y*, -*z*] is 87.22 (4)°, which is close to the ideal value of 90°. Nonclassical C—H···O hydrogen bonds (Table 1) occur in the structure, which link adjacent complex molecule into a three-dimensional network , Fig. 2 and Fig. 3.

### 2. Experimental

CoCl_2._2H_2_O (0.116 g,1 mmol) was dissolved in methanol (10 ml). To this solution, phenyacetic acid (C_6_H_5_CH_2_COOH; 0.136 g, 1 mmol) was added and the mixture was stirred for *ca* 10 min to obtain a bleu solution. 4-(Dimethylamino)pyridine (0.122 g, 1 mmol) was added and the mixture was stirred for an additional 2 h. Single crystals suitable for X-ray diffraction were obtained from a methanol solution of the title complex by slow evaporation.

### 3. Refinement

The C-bound H atoms were included in calculated positions, with C—H = 0.93 Å (aromatic), 0.96 Å (methyl), 0.97 Å(aliphatic) and refined using a riding model, with *U*_iso_(H) = 1.5*U*_eq_(C) for the methyl groups and 1.2*U*_eq_(C) for the remainder.

### Crystal data

**Table:**

[Co_2_(C_8_H_7_O_2_)_4_(C_7_H_10_N_2_)_2_]	*Z* = 1
*M**_r_* = 902.74	*F*(000) = 470
Triclinic, *P*1	*D*_x_ = 1.453 Mg m^−^^3^
Hall symbol: -P 1	Mo *K*α radiation, λ = 0.71073 Å
*a* = 8.107 (5) Å	Cell parameters from 4872 reflections
*b* = 11.043 (5) Å	θ = 2.3–25°
*c* = 12.573 (5) Å	µ = 0.86 mm^−^^1^
α = 99.766 (5)°	*T* = 150 K
β = 101.878 (5)°	Box, blue
γ = 105.335 (5)°	0.15 × 0.1 × 0.01 mm
*V* = 1031.9 (9) Å^3^	

### Data collection

**Table:**

Bruker APEXII CCD diffractometer	3645 independent reflections
Radiation source: fine-focus sealed tube	3352 reflections with *I* > 2σ(*I*))
Graphite monochromator	*R*_int_ = 0.019
ω and φ scans	θ_max_ = 25.0°, θ_min_ = 2.7°
Absorption correction: multi-scan (*SADABS*; Bruker, 2012)	*h* = −9→9
*T*_min_ = 0.902, *T*_max_ = 0.991	*k* = −13→13
9238 measured reflections	*l* = −14→14

### Refinement

**Table:**

Refinement on *F*^2^	0 restraints
Least-squares matrix: full	H-atom parameters constrained
*R*[*F*^2^ > 2σ(*F*^2^)] = 0.025	*w* = 1/[σ^2^(*F*_o_^2^) + (0.0206*P*)^2^ + 0.6871*P*] where *P* = (*F*_o_^2^ + 2*F*_c_^2^)/3
*wR*(*F*^2^) = 0.060	(Δ/σ)_max_ < 0.001
*S* = 1.05	Δρ_max_ = 0.25 e Å^−^^3^
3645 reflections	Δρ_min_ = −0.24 e Å^−^^3^
271 parameters	

### Special details

**Table:**

Geometry. All e.s.d.'s (except the e.s.d. in the dihedral angle between two l.s. planes) are estimated using the full covariance matrix. The cell e.s.d.'s are taken into account individually in the estimation of e.s.d.'s in distances, angles and torsion angles; correlations between e.s.d.'s in cell parameters are only used when they are defined by crystal symmetry. An approximate (isotropic) treatment of cell e.s.d.'s is used for estimating e.s.d.'s involving l.s. planes.
Refinement. Refinement of *F*^2^ against ALL reflections. The weighted *R*-factor *wR* and goodness of fit *S* are based on *F*^2^, conventional *R*-factors *R* are based on *F*, with *F* set to zero for negative *F*^2^. The threshold expression of *F*^2^ > σ(*F*^2^) is used only for calculating *R*-factors(gt) *etc*. and is not relevant to the choice of reflections for refinement. *R*-factors based on *F*^2^ are statistically about twice as large as those based on *F*, and *R*- factors based on ALL data will be even larger.

### Fractional atomic coordinates and isotropic or equivalent isotropic displacement parameters (Å^2^)

**Table:**

	*x*	*y*	*z*	*U*_iso_*/*U*_eq_	
Co1	0.56888 (3)	0.01135 (2)	0.114253 (18)	0.01603 (8)	
O1	0.70384 (17)	0.18802 (11)	0.09742 (10)	0.0256 (3)	
O3	0.75242 (16)	−0.06983 (12)	0.07678 (10)	0.0241 (3)	
O2	0.59969 (17)	0.17191 (12)	−0.08452 (10)	0.0248 (3)	
O4	0.64516 (17)	−0.08060 (13)	−0.10383 (10)	0.0277 (3)	
N1	0.7414 (2)	0.15583 (15)	0.63408 (12)	0.0259 (3)	
N2	0.64381 (19)	0.06270 (13)	0.28539 (11)	0.0178 (3)	
C10	0.7997 (2)	0.46694 (16)	0.12315 (14)	0.0192 (4)	
C3	0.7158 (2)	−0.00546 (16)	0.34964 (14)	0.0197 (4)	
H3	0.7415	−0.0764	0.3139	0.024*	
C11	0.7164 (3)	0.55861 (17)	0.10444 (16)	0.0256 (4)	
H11	0.6661	0.5599	0.0313	0.031*	
C1	0.7158 (2)	0.12779 (17)	0.52207 (14)	0.0195 (4)	
C18	0.8183 (2)	−0.25048 (16)	−0.17600 (14)	0.0181 (4)	
C13	0.7818 (3)	0.64805 (18)	0.30092 (16)	0.0302 (4)	
H13	0.7766	0.7085	0.3603	0.036*	
C17	0.8651 (2)	−0.18185 (17)	−0.05447 (14)	0.0205 (4)	
H17A	0.9855	−0.1236	−0.0338	0.025*	
H17B	0.8624	−0.2454	−0.0097	0.025*	
C23	0.9124 (2)	−0.20046 (17)	−0.24739 (15)	0.0222 (4)	
H23	1.0052	−0.1233	−0.2202	0.027*	
C2	0.7540 (2)	0.02208 (17)	0.46401 (14)	0.0216 (4)	
H2	0.8051	−0.0289	0.5033	0.026*	
C19	0.6790 (2)	−0.36483 (17)	−0.21909 (15)	0.0245 (4)	
H19	0.6135	−0.399	−0.1724	0.029*	
C9	0.8102 (3)	0.37074 (16)	0.02588 (15)	0.0236 (4)	
H9A	0.9324	0.3717	0.0358	0.028*	
H9B	0.7754	0.3982	−0.0424	0.028*	
C14	0.8655 (3)	0.55707 (19)	0.32101 (16)	0.0311 (4)	
H14	0.9163	0.5563	0.3942	0.037*	
C4	0.6139 (2)	0.16674 (17)	0.34079 (14)	0.0228 (4)	
H4	0.568	0.2179	0.299	0.027*	
C15	0.8741 (2)	0.46726 (18)	0.23306 (15)	0.0267 (4)	
H15	0.9303	0.4063	0.2476	0.032*	
C5	0.6466 (2)	0.20198 (17)	0.45452 (15)	0.0246 (4)	
H5	0.6231	0.2753	0.4876	0.029*	
C22	0.8696 (3)	−0.26441 (19)	−0.35918 (15)	0.0274 (4)	
H22	0.934	−0.23	−0.4062	0.033*	
C16	0.7435 (2)	−0.10501 (16)	−0.02568 (14)	0.0180 (4)	
C20	0.6361 (3)	−0.42857 (18)	−0.33028 (16)	0.0291 (4)	
H20	0.5425	−0.5053	−0.3579	0.035*	
C12	0.7061 (3)	0.64871 (18)	0.19226 (17)	0.0322 (5)	
H12	0.6487	0.7091	0.178	0.039*	
C8	0.6943 (2)	0.23260 (16)	0.01192 (14)	0.0186 (4)	
C7	0.8177 (3)	0.0810 (2)	0.70317 (16)	0.0328 (5)	
H7A	0.8256	0.1152	0.7802	0.049*	
H7B	0.9341	0.0858	0.6946	0.049*	
H7C	0.7438	−0.0076	0.6804	0.049*	
C21	0.7319 (3)	−0.37875 (19)	−0.40079 (15)	0.0292 (4)	
H21	0.7037	−0.4219	−0.4756	0.035*	
C6	0.6940 (3)	0.2633 (2)	0.68950 (16)	0.0353 (5)	
H6A	0.721	0.2693	0.7686	0.053*	
H6B	0.5694	0.2493	0.6611	0.053*	
H6C	0.7601	0.3421	0.6754	0.053*	

### Atomic displacement parameters (Å^2^)

**Table:**

	*U*^11^	*U*^22^	*U*^33^	*U*^12^	*U*^13^	*U*^23^
Co1	0.02007 (13)	0.01633 (13)	0.01213 (12)	0.00678 (9)	0.00475 (9)	0.00211 (9)
O1	0.0329 (7)	0.0188 (6)	0.0213 (7)	0.0030 (5)	0.0044 (6)	0.0066 (5)
O3	0.0262 (7)	0.0258 (7)	0.0218 (7)	0.0115 (5)	0.0093 (5)	0.0010 (5)
O2	0.0293 (7)	0.0213 (6)	0.0190 (6)	0.0020 (5)	0.0056 (6)	0.0022 (5)
O4	0.0278 (7)	0.0354 (8)	0.0261 (7)	0.0202 (6)	0.0074 (6)	0.0073 (6)
N1	0.0366 (9)	0.0275 (8)	0.0140 (7)	0.0111 (7)	0.0067 (7)	0.0044 (6)
N2	0.0212 (7)	0.0178 (7)	0.0146 (7)	0.0072 (6)	0.0048 (6)	0.0029 (6)
C10	0.0187 (9)	0.0152 (8)	0.0212 (9)	0.0001 (7)	0.0075 (7)	0.0034 (7)
C3	0.0234 (9)	0.0163 (8)	0.0209 (9)	0.0086 (7)	0.0065 (7)	0.0030 (7)
C11	0.0319 (10)	0.0201 (9)	0.0238 (9)	0.0060 (8)	0.0054 (8)	0.0080 (8)
C1	0.0196 (9)	0.0213 (9)	0.0160 (8)	0.0035 (7)	0.0054 (7)	0.0040 (7)
C18	0.0181 (8)	0.0187 (9)	0.0209 (9)	0.0111 (7)	0.0059 (7)	0.0041 (7)
C13	0.0418 (12)	0.0204 (9)	0.0283 (10)	0.0084 (9)	0.0156 (9)	0.0003 (8)
C17	0.0190 (9)	0.0212 (9)	0.0213 (9)	0.0081 (7)	0.0052 (7)	0.0023 (7)
C23	0.0182 (9)	0.0231 (9)	0.0252 (9)	0.0068 (7)	0.0047 (7)	0.0066 (8)
C2	0.0265 (10)	0.0217 (9)	0.0196 (9)	0.0109 (8)	0.0055 (7)	0.0085 (7)
C19	0.0248 (10)	0.0238 (9)	0.0256 (10)	0.0062 (8)	0.0090 (8)	0.0068 (8)
C9	0.0301 (10)	0.0185 (9)	0.0221 (9)	0.0041 (8)	0.0114 (8)	0.0047 (7)
C14	0.0390 (12)	0.0316 (11)	0.0200 (9)	0.0107 (9)	0.0046 (9)	0.0034 (8)
C4	0.0330 (10)	0.0216 (9)	0.0180 (9)	0.0151 (8)	0.0061 (8)	0.0057 (7)
C15	0.0289 (10)	0.0255 (10)	0.0270 (10)	0.0132 (8)	0.0043 (8)	0.0059 (8)
C5	0.0346 (11)	0.0213 (9)	0.0199 (9)	0.0144 (8)	0.0069 (8)	0.0016 (7)
C22	0.0283 (10)	0.0366 (11)	0.0224 (10)	0.0131 (9)	0.0097 (8)	0.0117 (8)
C16	0.0165 (8)	0.0139 (8)	0.0234 (9)	0.0029 (7)	0.0081 (7)	0.0030 (7)
C20	0.0277 (10)	0.0222 (9)	0.0285 (10)	0.0035 (8)	0.0000 (8)	−0.0017 (8)
C12	0.0420 (12)	0.0205 (10)	0.0393 (12)	0.0154 (9)	0.0124 (10)	0.0098 (9)
C8	0.0189 (9)	0.0179 (9)	0.0208 (9)	0.0070 (7)	0.0089 (7)	0.0026 (7)
C7	0.0410 (12)	0.0410 (12)	0.0191 (10)	0.0146 (10)	0.0071 (9)	0.0124 (9)
C21	0.0347 (11)	0.0350 (11)	0.0169 (9)	0.0160 (9)	0.0022 (8)	0.0006 (8)
C6	0.0547 (14)	0.0327 (11)	0.0196 (10)	0.0140 (10)	0.0148 (9)	0.0022 (8)

### Geometric parameters (Å, º)

**Table:**

Co1—O3	2.0224 (15)	C13—H13	0.93
Co1—O1	2.0382 (14)	C17—C16	1.519 (2)
Co1—O2^i^	2.0429 (15)	C17—H17A	0.97
Co1—N2	2.0460 (16)	C17—H17B	0.97
Co1—O4^i^	2.0628 (16)	C23—C22	1.388 (3)
Co1—Co1^i^	2.8020 (12)	C23—H23	0.93
O1—C8	1.253 (2)	C2—H2	0.93
O3—C16	1.262 (2)	C19—C20	1.381 (3)
O2—C8	1.256 (2)	C19—H19	0.93
O2—Co1^i^	2.0429 (15)	C9—C8	1.524 (2)
O4—C16	1.247 (2)	C9—H9A	0.97
O4—Co1^i^	2.0628 (16)	C9—H9B	0.97
N1—C1	1.349 (2)	C14—C15	1.381 (3)
N1—C6	1.453 (2)	C14—H14	0.93
N1—C7	1.454 (2)	C4—C5	1.366 (2)
N2—C3	1.343 (2)	C4—H4	0.93
N2—C4	1.345 (2)	C15—H15	0.93
C10—C11	1.383 (3)	C5—H5	0.93
C10—C15	1.388 (3)	C22—C21	1.380 (3)
C10—C9	1.510 (2)	C22—H22	0.93
C3—C2	1.369 (2)	C20—C21	1.384 (3)
C3—H3	0.93	C20—H20	0.93
C11—C12	1.387 (3)	C12—H12	0.93
C11—H11	0.93	C7—H7A	0.96
C1—C2	1.410 (2)	C7—H7B	0.96
C1—C5	1.411 (2)	C7—H7C	0.96
C18—C23	1.386 (2)	C21—H21	0.93
C18—C19	1.388 (3)	C6—H6A	0.96
C18—C17	1.506 (2)	C6—H6B	0.96
C13—C12	1.380 (3)	C6—H6C	0.96
C13—C14	1.382 (3)		
			
O3—Co1—O1	93.27 (6)	C3—C2—H2	120.1
O3—Co1—O2^i^	87.33 (6)	C1—C2—H2	120.1
O1—Co1—O2^i^	163.77 (5)	C20—C19—C18	120.91 (17)
O3—Co1—N2	102.92 (6)	C20—C19—H19	119.5
O1—Co1—N2	94.29 (5)	C18—C19—H19	119.5
O2^i^—Co1—N2	101.39 (5)	C10—C9—C8	114.21 (14)
O3—Co1—O4^i^	163.73 (5)	C10—C9—H9A	108.7
O1—Co1—O4^i^	85.64 (6)	C8—C9—H9A	108.7
O2^i^—Co1—O4^i^	89.27 (7)	C10—C9—H9B	108.7
N2—Co1—O4^i^	93.35 (5)	C8—C9—H9B	108.7
O3—Co1—Co1^i^	89.34 (4)	H9A—C9—H9B	107.6
O1—Co1—Co1^i^	79.04 (4)	C15—C14—C13	120.40 (18)
O2^i^—Co1—Co1^i^	84.75 (4)	C15—C14—H14	119.8
N2—Co1—Co1^i^	166.44 (4)	C13—C14—H14	119.8
O4^i^—Co1—Co1^i^	74.50 (4)	N2—C4—C5	124.16 (16)
C8—O1—Co1	129.17 (11)	N2—C4—H4	117.9
C16—O3—Co1	116.62 (11)	C5—C4—H4	117.9
C8—O2—Co1^i^	121.65 (11)	C14—C15—C10	120.78 (17)
C16—O4—Co1^i^	133.30 (12)	C14—C15—H15	119.6
C1—N1—C6	120.56 (16)	C10—C15—H15	119.6
C1—N1—C7	121.55 (16)	C4—C5—C1	120.39 (16)
C6—N1—C7	117.89 (15)	C4—C5—H5	119.8
C3—N2—C4	115.61 (15)	C1—C5—H5	119.8
C3—N2—Co1	124.00 (11)	C21—C22—C23	120.28 (18)
C4—N2—Co1	120.31 (11)	C21—C22—H22	119.9
C11—C10—C15	118.12 (17)	C23—C22—H22	119.9
C11—C10—C9	120.35 (16)	O4—C16—O3	125.35 (16)
C15—C10—C9	121.52 (16)	O4—C16—C17	118.12 (15)
N2—C3—C2	124.67 (16)	O3—C16—C17	116.52 (15)
N2—C3—H3	117.7	C19—C20—C21	120.23 (18)
C2—C3—H3	117.7	C19—C20—H20	119.9
C10—C11—C12	121.52 (18)	C21—C20—H20	119.9
C10—C11—H11	119.2	C13—C12—C11	119.52 (18)
C12—C11—H11	119.2	C13—C12—H12	120.2
N1—C1—C2	122.71 (16)	C11—C12—H12	120.2
N1—C1—C5	122.05 (16)	O1—C8—O2	125.30 (16)
C2—C1—C5	115.23 (16)	O1—C8—C9	117.19 (15)
C23—C18—C19	118.53 (16)	O2—C8—C9	117.50 (15)
C23—C18—C17	120.81 (16)	N1—C7—H7A	109.5
C19—C18—C17	120.66 (16)	N1—C7—H7B	109.5
C12—C13—C14	119.66 (18)	H7A—C7—H7B	109.5
C12—C13—H13	120.2	N1—C7—H7C	109.5
C14—C13—H13	120.2	H7A—C7—H7C	109.5
C18—C17—C16	114.52 (14)	H7B—C7—H7C	109.5
C18—C17—H17A	108.6	C22—C21—C20	119.40 (17)
C16—C17—H17A	108.6	C22—C21—H21	120.3
C18—C17—H17B	108.6	C20—C21—H21	120.3
C16—C17—H17B	108.6	N1—C6—H6A	109.5
H17A—C17—H17B	107.6	N1—C6—H6B	109.5
C18—C23—C22	120.65 (17)	H6A—C6—H6B	109.5
C18—C23—H23	119.7	N1—C6—H6C	109.5
C22—C23—H23	119.7	H6A—C6—H6C	109.5
C3—C2—C1	119.85 (16)	H6B—C6—H6C	109.5

Symmetry code: (i) −*x*+1, −*y*, −*z*.

### Hydrogen-bond geometry (Å, º)

**Table:**

*D*—H···*A*	*D*—H	H···*A*	*D*···*A*	*D*—H···*A*
C6—H6*A*···O2^ii^	0.96	2.53	3.337 (3)	142
C23—H23···O3^iii^	0.93	2.58	3.469 (3)	159

Symmetry codes: (ii) *x*, *y*, *z*+1; (iii) −*x*+2, −*y*, −*z*.
